# Human-to-Dog Transmission of Methicillin-Resistant *Staphylococcus aureus*

**DOI:** 10.3201/eid1508.081635

**Published:** 2009-08

**Authors:** Bronwyn E. Rutland, J. Scott Weese, Carole Bolin, Jennifer Au, Anurag N. Malani

**Affiliations:** Michigan State University, East Lansing, Michigan, USA (B.E. Rutland, C. Bolin, J. Au); University of Guelph, Guelph, Ontario, Canada (J.S. Weese); St. Joseph Mercy Health System, Ann Arbor, Michigan, USA (A.N. Malani)

**Keywords:** Dogs, companion animals, humans, antimicrobial resistance, MRSA, interspecies transmission, bacteria, letter

**To the Editor:** In November 2007, a 76-year-old man with diabetes mellitus, chronic lymphocytic leukemia, and chronic obstructive pulmonary disease, who was being treated with prolonged corticosteroid therapy, received a diagnosis of invasive pulmonary aspergillosis. After 4 weeks of voriconazole therapy, cellulitis with substantial erythema, induration, and tenderness developed in his right bicep muscle. Bacterial cultures from a skin biopsy sample yielded methicillin-resistant *Staphylococcus aureus* (MRSA), resistant to trimethoprim/sulfamethoxazole, clindamycin, erythromycin, tetracycline, and ciprofloxacin. The patient received intravenous vancomycin for 3 weeks. After prolonged hospitalization, he was discharged but again hospitalized in February 2008 for cellulitis in the right ankle. Cultures of drainage around the ankle grew MRSA with a susceptibility pattern identical to that of the previous isolate. In April 2008, after the patient had received vancomycin for 1 week and the infection had resolved, a nasal swab showed carriage of MRSA with a susceptibility pattern identical to that of the previous isolates.

In late February 2008, the man’s 8-year-old spayed female Labrador retriever was examined for cellulitis and generalized abscessation of the neck area, which had not responded to empirical treatment with oral cephalexin. In December 2007, she had undergone surgery for a ruptured cranial cruciate ligament (right tibial plateau-leveling osteotomy). She had chewed some sutures out after surgery, and cultures of a purulent discharge from the incision grew *Pseudomonas aeruginosa*; this infection was successfully treated with enrofloxacin. As a result of implant failure, surgery was repeated in early February 2008. Cultures of the joint fluid and implants at this time were negative.

Physical examination in late February showed a large, firm area of extensive swelling on the ventral aspect of the dog’s neck and purulent discharge from ulcerations (Figure). She had dried discharge around her right stifle and was moderately lame on that leg. Cultures of blood, tissue samples from her neck, and fluid draining from the right stifle joint all grew MRSA (also resistant to trimethoprim/sulfamethoxazole, clindamycin, erythromycin, tetracycline, enrofloxacin, marbofloxacin, and orbifloxacin). A biopsy sample of the neck showed severe, acute, multifocal, neutrophilic vasculitis with numerous fibrin thrombi and moderate to severe, superficial to deep, perivascular to periadnexal, suppurative lymphohistiocytic dermatitis. The dog became increasingly lethargic; systemic inflammatory response syndrome developed, and the neck became severely ulcerated and necrotic. Aggressive therapy with intravenous hydration and antimicrobial drugs (clindamycin and cefazolin, given before culture results were known) produced little clinical response. Extensive regions of skin sloughed, the face and neck became edematous, and focal masses developed within the lips. Signs of septic shock developed, and the dog was humanely euthanized 48 hours after this admission for skin lesions.

**Figure:**
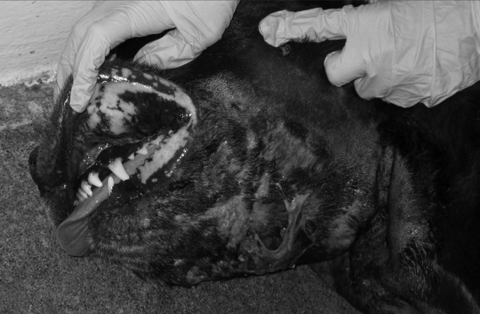
Extensive neck swelling with ulcerations and purulent discharge on 8-year-old spayed female Labrador retriever. Culture of the exudate and a macerated skin biopsy specimen grew methicillin-resistant *Staphylococcus aureus.*

Pulsed-field gel electrophoresis was performed on isolates from the man and the dog. The isolates were indistinguishable and not consistent with recognized USA epidemic clones. *Spa* typing was also performed, and all isolates were *spa* type 3, also known as t037 according to the Ridom classification. Genes encoding for production of the Panton-Valentine leukocidin gene were not detected by real-time PCR (*1*).

Prevalence of MRSA in humans is increasing in most of the world. Similarly, MRSA colonization and infections in pets have increased in the past few years (*1*–*5*). MRSA can be transmitted between persons and their pets (*1*–*4*,*6*), although the route of transmission, risk factors for transmission, and incidence of interspecies transmission are not well understood. We describe a case of human-to-dog transmission of MRSA, which led to euthanasia of the dog.

Given the degree of antimicrobial-drug resistance in the MRSA isolates and the close ongoing contact of the human with the healthcare system, we suspect that the source of the MRSA infection was the human healthcare system. The dog likely acquired MRSA through close contact with her owner in that she had an open wound from complications of her orthopedic surgery. Most cases of MRSA in dogs have been associated with colonization, skin and soft tissue infections, or surgical site infections (*1*,*7*,*8*). Human-to-dog transmission is also supported by the temporal association of both infections and the dog’s negative bacterial cultures in early February.

Studies of MRSA infection and colonization in household pets show that pets tend to be infected or colonized with MRSA strain types from the local human population (*3*,*7*). We, along with others, believe that MRSA in pets is closely linked to MRAS in humans and that infected or colonized humans may often be the source of MRSA in household animals (*5*). For some MRSA cases, infection reportedly resolved after the reservoir (either humans or animals in the household) was identified and treated appropriately (*4*–*6*). With MRSA infections reaching epidemic proportions, physicians and veterinarians must be aware of MRSA and the risk for cross-infection between species. To help develop infection control and treatment strategies to reduce the risk for infection within a household, further study is needed to clarify the epidemiology of interspecies transmission of MRSA.
